# miR‐15b enhances the proliferation and migration of lung adenocarcinoma by targeting BCL2

**DOI:** 10.1111/1759-7714.13382

**Published:** 2020-03-27

**Authors:** Jun Wang, Shupeng Yao, Yanping Diao, Yan Geng, Yanling Bi, Guangyue Liu

**Affiliations:** ^1^ Department of Thoracic Surgery Yantai Affiliated Hospital of Binzhou Medical University Yantai China; ^2^ Department of Respiratory Medicine Liaocheng Dongchangfu Peopleʼs Hospital Liaocheng China; ^3^ Department of Gastrointestinal Surgery the Peopleʼs Hospital of Zhangqiu Area Jinan China; ^4^ Operation Room The Peopleʼs Hospital of Zhangqiu Area Jinan China; ^5^ Department of Outpatient Weifang Peopleʼs Hospital Weifang China

**Keywords:** BCL2, EMT, lung adenocarcinoma (LUAD), miR‐15b, proliferation

## Abstract

**Background:**

Lung adenocarcinoma (LUAD) is a subtype of lung cancer (LC), which is the most common tumor worldwide. Accumulating evidence has elucidated an important role of microRNAs (miRNAs) in mediating the development and progression of several tumors. The purpose of this study was to explore the role and underlying mechanism of miR‐15b in LUAD.

**Methods:**

CCK‐8 and Transwell assays were conducted to measure the capacities of cell viability and migration in SPC‐A1 cells. Luciferase assay was utilized to verifymiR‐15b direct binding to BCL2 mRNA 3′‐UTR.

**Results:**

We determined that miR‐15b was overexpressed in LUAD and miR‐15b overexpression predicted a significantly worse outcome in patients with LUAD. miR‐15b improved LUAD growth in vitro and vivo. miR‐15b enhanced cell migration and epithelial–mesenchymal transition (EMT) in LUAD. miR‐15b promoted cell viability, migration and EMT through inhibiting BCL2 expression by targeting to its mRNA 3′‐UTR. BCL2 reversed functions of miR‐15b on promoting cell proliferation, migration and EMT in SPC‐A1 cells.

**Conclusions:**

miR‐15b promoted cell viability, migration and EMT by targeting BCL2 in LUAD. The newly identified miR‐15b/BCL2 axis provides a novel insight into the pathogenesis of LUAD.

## Introduction

Lung cancer (LC) is the leading cause of cancer‐related mortality worldwide, with lung adenocarcinoma (LUAD) being the most frequently diagnosed histological subtype.^1^, ^2^ Most patients with LUAD are usually diagnosed at advanced stages and have a poor prognosis.^3^, ^4^ It is therefore necessary to define new molecular biomarkers for the early diagnosis of LUAD.

MicroRNAs (miRNAs) are endogenous non‐coding RNAs containing 18–25 nucleotides.^5^ MiRNAs mediate the repression of translation to regulate the expression of target genes at post‐transcriptional levels.^6^, ^7^ In this way, miRNAs may play important roles in several processes that include adhesion, differentiation, cell death and metastasis.^8^, ^9^ MiR‐15b was upregulated and acted as an oncogene in multiple diseases, including hepatocellular carcinoma, ovarian cancer and chronic neuropathic pain.^10^, ^11^, ^12^ MiR‐15b facilitated tumorigenicity by enhancing growth and invasiveness, and predicted tumor recurrence in prostate cancer.^13^ MiR‐15b may act as an independent risk factor that has been associated with a poor outcome for cervical carcinoma patients.^14^ However, miR‐15b acted as a tumor suppressor to inhibit cell proliferation of osteosarcoma.^15^ The expression and the regulatory mechanism is still unclear in LUAD.

BCL2 apoptosis regulator (BCL2) encodes an integral outer mitochondrial membrane which could block apoptosis of several cells such as lymphocytes.^16^ BCL2‐family expression profile has been reported to be related to tumor aggressiveness in B‐cell malignancies.^17^ El Shazly *et al*. indicated that BCL2 acted as mRNA biomarkers for monitoring the immune response in critically ill children.^18^ What is more, BCL2 transcriptional alterations in umbilical‐cord blood cells can be putative biomarkers for obesity.^19^ Dysregulation expression of BCL2 has been reported to promote multiple classes of mature non‐hodgkin B cell lymphoma in mice.^20^ In addition, the expression of BCL2 is associated with prognosis in primary central nervous system diffuse large B‐cell lymphoma, and even in invasive breast cancer.^21^, ^22^

Epithelial‐mesenchymal transition (EMT) refers to the biological process by which epithelial cells are transformed into mesenchymal phenotype cells by a specific process.^23^ The main features of EMT are the reduction of E‐cadherin expression, increase of N‐cadherin expression, and the transformation of cytokeratin cytoskeleton into vimentin‐based cytoskeleton.^24^, ^25^ EMT is an important biological process by which epithelial‐derived malignant tumor cells acquire the ability to migrate and invade.^26^, ^27^ To elucidate the molecular mechanism of regulating EMT in malignant tumor cells is a key scientific issue in the study of EMT mechanisms.

## Methods

### Patients and tissue specimens

A total of 46 LUAD patients were selected from Yantai Affiliated Hospital of Binzhou Medical University during 2016 and 2018, and paired LUAD and adjacent normal lung tissues were obtained. All patients had not been treated with radiotherapy or chemotherapy prior to surgery. The tissues were snap‐frozen in liquid nitrogen and stored at −80°C. This study was approved by the Ethics Committee of Yantai Affiliated Hospital of Binzhou Medical University. All patients provided their written informed consent.

### Cell culture and transfection

The human LUAD cells PC9/ZD and SPC‐A1 cells, and bronchial epithelial cells MRC‐5 were purchased from the American Type Culture Collection (ATCC) (Manassas, VA, USA). All the cells were cultured in Dulbecco's modified Eagle's medium (DMEM: Gibco; Thermo Fisher Scientific, Waltham, MA, USA) and supplemented with 10% FBS (Gibco; Thermo Fisher Scientific) at 37°C in 5% CO_2_ with a humidified atmosphere of 95% air.

The miR‐15b mimic and miR‐15b inhibitor were purchased from GenePharma (Shanghai, China). BCL2 overexpressed plasmids (pcDNA3.1‐BCL2) obtained from the Chinese Academy of Sciences (Changchun, China). SPC‐A1 cells were utilized to perform the transfection using Lipofectamine 2000 (Invitrogen, Carlsbad, CA, USA).

### Western blot analysis

SPC‐A1 cells were lysed with RIPA lysis buffer (Beyotime, Shanghai, China), and bicinchoninic acid (BCA) (Thermo Fisher Scientific; Waltham, MA, USA) was employed to measure the protein concentration. Protein samples were separated by 10% sodium dodecyl sulfate polyacrylamide gel (SDS‐PAGE) and transferred onto polyvinylidenedifluoride (PVDF) membrane (Millipore, Billerica, MA, USA). Subsequently, 5% skim milk was utilized to block the membrane for 2 hours, and the membrane was then incubated with primary antibodies overnight at 4°C, including BCL2, EMT markers (E‐cadherin and N‐cadherin) and GAPDH. Next, corresponding secondary antibodies were incubated in the membrane, and ECL (Pierce, Rockford, IL, USA) was then applied to measure the protein expression levels.

### Quantitative real‐time polymerase chain reaction (qRT‐PCR)

A TRIzol reagent (Invitrogen, Carlsbad, CA, USA) was utilized to extract total RNAs from tissues and cells. The first‐strand cDNA synthesis kit (Promega, Madison, WI, USA) was used to perform the reverse transcription and synthesize the cDNA. Next, the SYBR Green Master mix kit (TaKaRa, Otsu, Shiga, Japan) was employed to carry out the qRT‐PCR on ABI Prizm 7300 Sequence Detection System (Applied Biosystems, Foster City, CA, USA). The primers were: miR‐15b forward, 5′‐TAGCAGCACATAATGGTTTGTG‐3′; reverse, 5′‐GCGTAGCAGCACATCATGG‐3′; U6 forward, 5′‐CTCGCTTCGGCAGCACA‐3′, reverse, 5′‐AACGCTTCACGAATTTGCGT‐3′; BCL2 forward, 5′‐TGGACAACCATGACCTTGGAC‐3′,reverse, 5′‐GTGCTCAGCTTGGTATGCAGAA‐3′; GAPDH forward, 5′‐CGGAGTCAACGGATTTGGTCGTAT‐3′, reverse, 5′‐AGCCTTCTCCATGGTGGTGAAGAC‐3′. The U6 and GAPDH were utilized as the normalization of miR‐15b and BCL2 and quantified by the 2^‐∆∆Ct^ method.

### Transwell assay

Transwell inserts were placed into a 24‐well tissue culture plate to assess the migratory ability of SPC‐A1 cells. SPC‐A1 cells suspended in PBS free medium were seeded in the upper chamber. Meanwhile, fresh medium containing 20% FBS was added to the lower chamber, which was utilized to induce SPC‐A1 cell migration. After incubation for 24 hours, the unmigrated cells were removed using cotton swabs. The migrated cells were fixed using methanol and then stained with crystal violet. Finally, an inverted microscope (Olympus Corporation, Tokyo, Japan) was used to count the number of cells.

### Wound healing assay

A wound‐healing assay was used to evaluate the migration ability. Briefly, SPC‐A1 cells were seeded into six‐well plates and allowed to grow until there was a 90% confluence. A sterile pipette tip was used to generate wounds on the bottom of each well. The plates were rinsed three times with PBS to remove the detached cells, and then further incubated with serum‐free medium under the same conditions for 24 hours. Cells were photographed at 0 and 24 hours using a microscope (Leica, Wetzlar, Germany).

### Cell Counting Kit‐8 (CCK‐8) assay

CCK‐8 assay (Dojindo Molecular Technologies, Kumamoto, Japan) was conducted to evaluate the cell proliferation. Specific cells were seeded into 96‐well plates, and cultured for 24, 48, 72 or 96 hours at 37°C with 5% CO_2_. Subsequently, 10 μl CCK‐8 reagent was added to each well and incubated for 2 hours. The absorbance at a wave length of 450 nm was measured using an ELISA reader (Bio‐Rad Laboratories, Hercules, CA, USA).

### Dual luciferase assay

Cells were seeded into 24‐well plates and cultured to a confluence of 70% at 37°C. Together with miR‐15b mimic, pGL3‐BCL2‐WT or pGL3‐BCL2‐MUT vectors (Promega Corporation, Madison, WI, USA) were cotransfected into SPC‐A1 cells. The cells were harvested and lysed to perform luciferase assays after transfection for 48 hours. A firefly luciferase activity was calculated using a dual‐luciferase reporter assay system (Promega, Madison, WI, USA), with Renilla luciferase activity as the normalization.

### Statistical analysis

Data are expressed as mean ± SD. SPSS 19.0 (IBM, Armonk, NY, USA) and Graphpad Prism6 (La Jolla, CA, USA) were employed to perform the statistical analysis. Kaplan‐Meier method log‐rank test was used to evaluate the five‐year overall survival of LUAD patients. *P* < 0.05 was considered statistically significant.

## Results

### Overexpression of miR‐15b associated with poor prognosis

**Figure 1:**
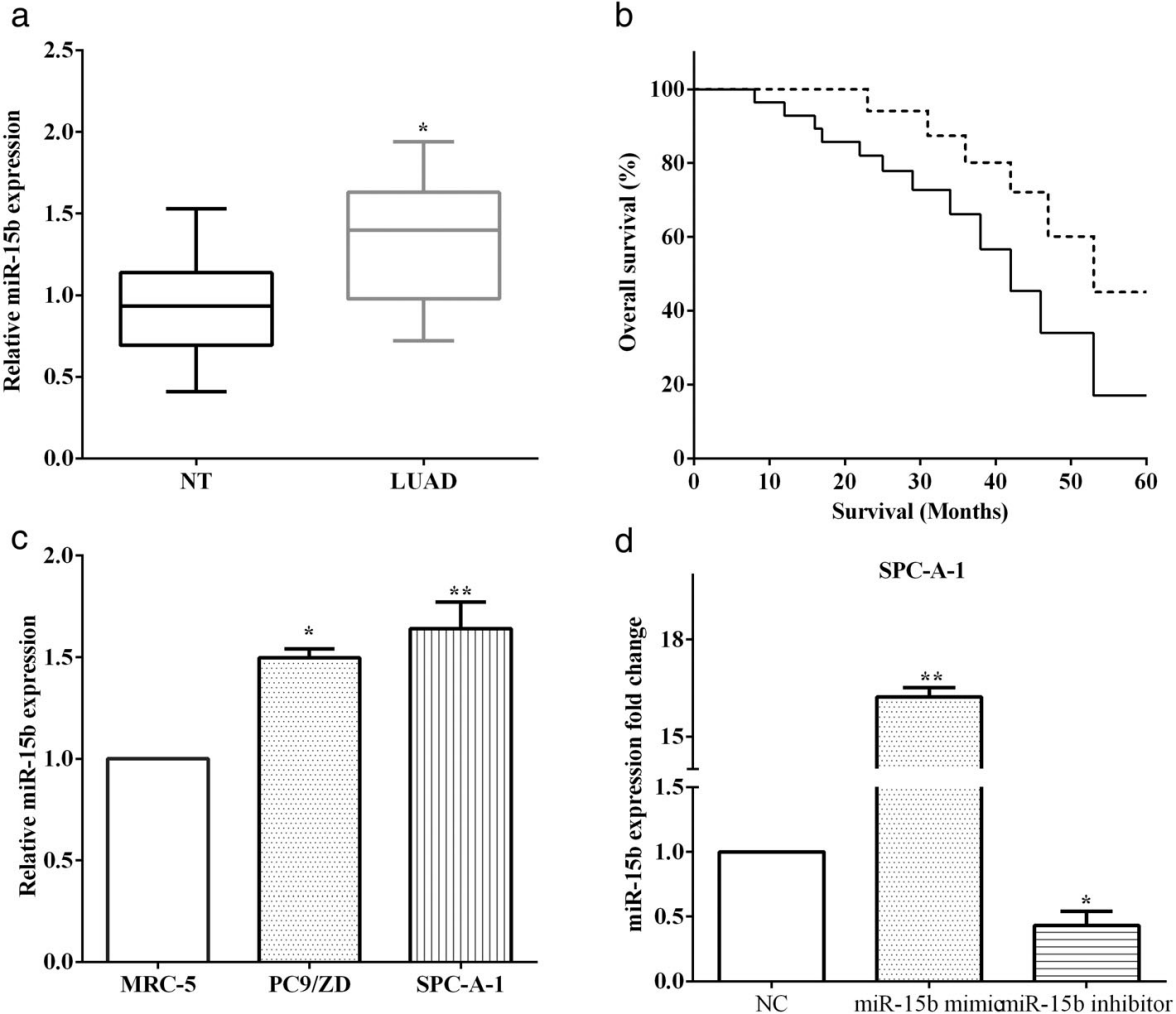
Overexpression of miR‐15b was associated with poor prognosis. (**a**) MiR‐15b was overexpressed in LUAD tissues versus adjacent normal tissues. (**b**) Overexpression of miR‐15b was association with worse overall survival in LUAD patients. (**c**) The expression of miR‐15b was lower in bronchial epithelial cell MRC‐5 than that in LUAD cell lines PC9/ZD and SPC‐A1. (**d**) The miR‐15b mimic or miR‐15b inhibitor was transfected in SPC‐A1 cells. (**b**) (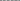) miR‐15b(−), (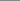) miR‐15b(+); (**c**) (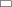) MRC‐5, (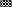) PC9/ZD, (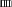) SPC‐A‐1; (**d**) (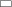) NC, (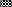) miR‐15b mimic, (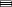) miR‐15b inhibitor.

The expression of miR‐15b was assessed using RT‐qPCR in LUAD and matched adjacent normal tissues. We discovered that miR‐15b was overexpressed in LUAD tissues versus adjacent normal tissues (*P* < 0.05) (Fig 1(a)). To verify the association between the expression of miR‐15b and overall survival of LUAD patients, we plotted the survival curve using the Kaplan‐Meier method. As demonstrated in Fig 1b, overexpression of miR‐15b was associated with worse overall survival of LUAD patients (*P* < 0.05) (Fig 1b). The expression of miR‐15b in LUAD cells PC9/ZD and SPC‐A1 cells, and bronchial epithelial cells (MRC‐5) were evaluated by RT‐qPCR, and expression of miR‐15b was lower in bronchial epithelial cell MRC‐5 than that in LUAD cell lines PC9/ZD (*P* < 0.05) and SPC‐A1 (*P* < 0.01) (Fig 1c). To explore the important roles of miR‐15b in LUAD, the miR‐15b mimic or the miR‐15b inhibitor was transfected in SPC‐A1 cells, to up‐ or downregulate the expression of miR‐15b (Fig 1d).

### MiR‐15b promoted LUAD growth in vitro and in vivo

**Figure 2:**
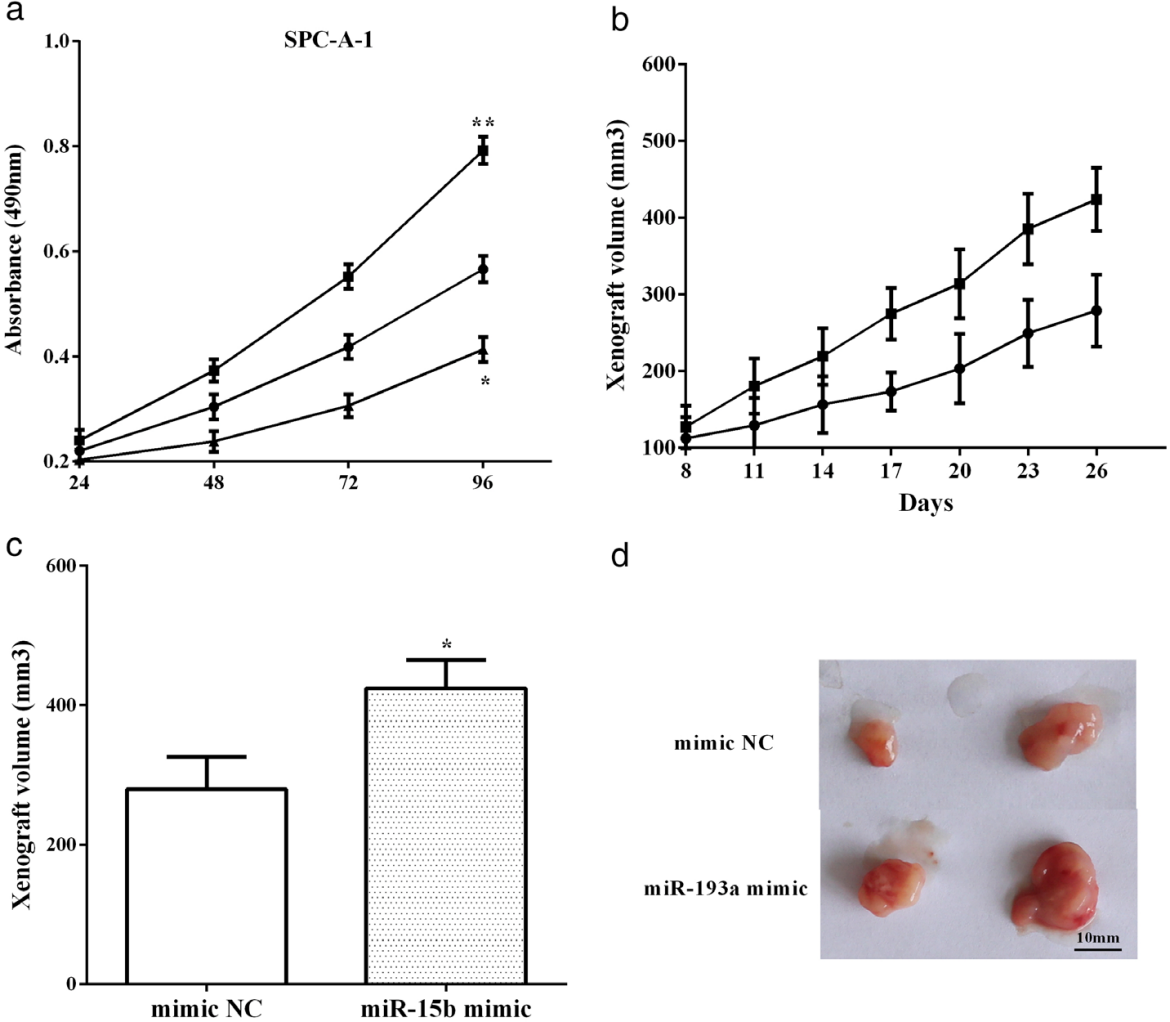
MiR‐15b promoted lung adenocarcinoma (LUAD) growth in vitro and in vivo. (**a**) The miR‐15b mimic improved cell proliferation, whereas miR‐15b inhibitor reduced the proliferative capacity in SPC‐A1 cells. (**b**) The group of transfecting control group had a slower growth rate than the miR‐15b mimic group. (**c**) The tumor volumes of cells transfected miR‐15b mimic were greater than the control group. (**a**) (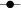) NC, (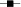) miR‐15b mimic, (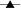) miR‐15b inhibitor; (**b**) (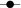) mimic NC, (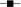) miR‐15b mimic, (**c**) (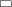) mimic NC, (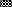) miR‐15b mimic.

The effect of miR‐15b on the proliferative and migratory abilities was assessed by CCK‐8 assay. The miR‐15b mimic improved cell proliferation (*P* < 0.05), whereas the miR‐15b inhibitor reduced the proliferative (*P* < 0.05) capacity in SPC‐A1 cells (Fig 2a). SPC‐A1 cells stably transfected with miR‐15b mimic or control plasmid were subcutaneously injected into the nude mice. The length and width of the xenografts were measured every three days after which the xenograft growth curve was drawn. As expected, the transfected control group had a slower growth rate than the miR‐15b mimic group (Fig 2b). After culture for 26 days, the nude mice were dissected and tumor volumes calculated. We discovered that the tumor volumes of cells transfected with the miR‐15b mimic were greater than the control group, which indicated that overexpression of miR‐15b promoted in vivo LUAD growth (*P* < 0.05) (Fig 2c).

### MiR‐15b enhanced cell migration and EMT in LUAD

**Figure 3:**
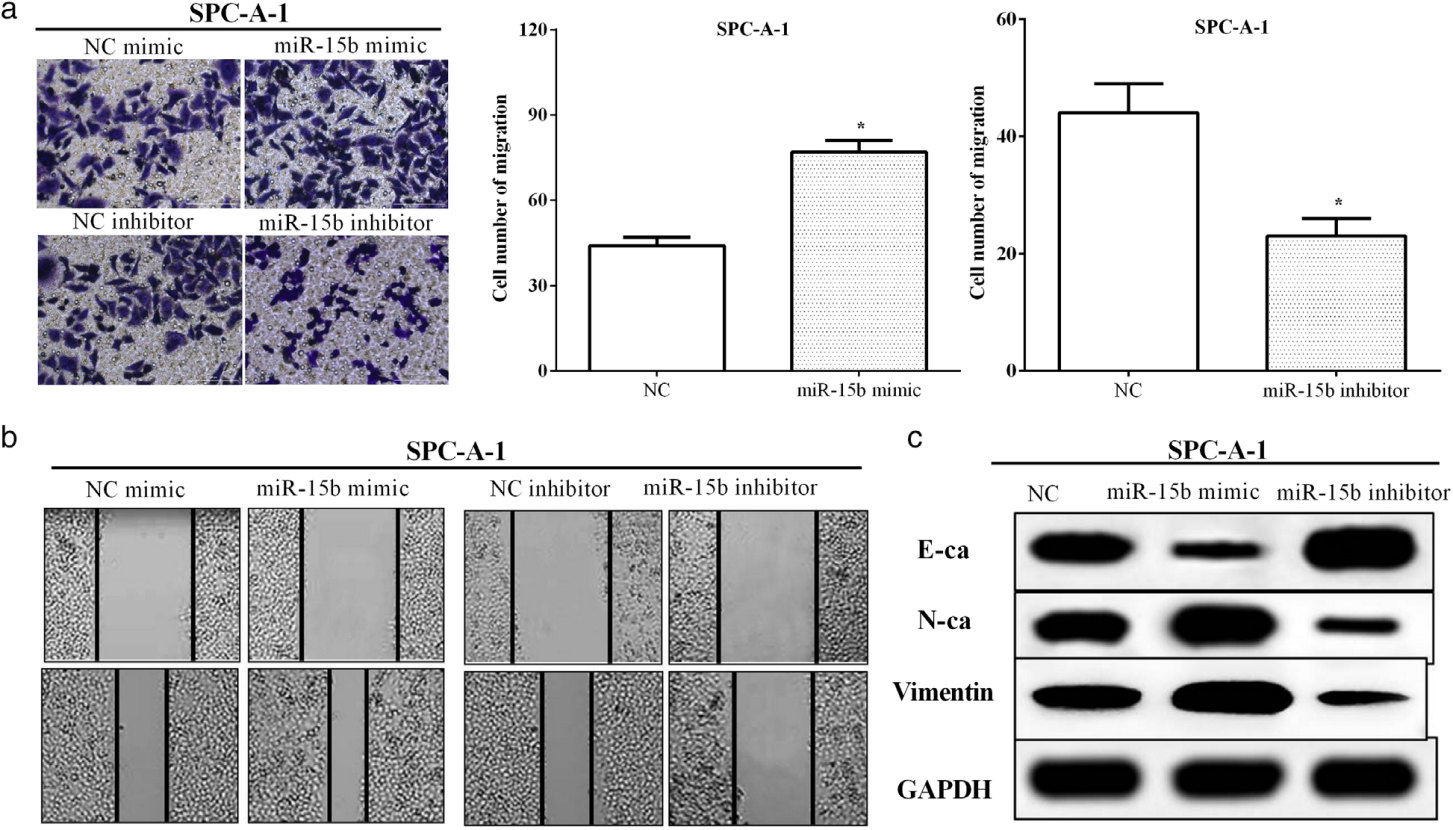
MiR‐15b enhanced cell migration and epithelial‐mesenchymal transition (EMT) in LUAD. (**a**) Transwell assay revealed that miR‐15b mimic promoted cell migration, while miR‐15b inhibitor reduced cell migration in APC‐A1 cells. (**b**) Wound healing assay indicated that cell migration was regulated by miR‐15b in APC‐A1 cells. (**c**) MiR‐15b promoted the EMT ability in LUAD cells.

Transwell assay was used to measure the cell migration after transfection of the miR‐15b mimic or the miR‐15b inhibitor in APC‐A1 cells. As expected, transfection of the miR‐15b mimic in APC‐A1 cells promoted cell migration (*P* < 0.05), while miR‐15b inhibitor reduced cell migration (*P* < 0.05) (Fig 3a). Wound healing assay was also used to calculate migratory ability. Figure 3b shows that the wound healing area of the NC groups was significantly greater than that in the miR‐15b mimic group. On the contrary, the wound area of NC was smaller than the miR‐15b group (Fig 3b). In addition, western blot analysis was utilized to measure the EMT protein expression, including E‐cadherin, N‐cadherin and vimentin. As expected, miR‐15b mimic enhanced the expression of N‐cadherin and vimentin while inhibited E‐cadherin expression. However, N‐cadherin and vimentin expression was decreased by miR‐15b inhibitor, while E‐cadherin expression was increased by miR‐15b inhibitor in APC‐A1 cells (Fig 3c), suggesting that miR‐15b enhanced the EMT ability in LUAD. These results indicated that miR‐15b was involved in the metastasis of LUAD cells.

### BCL2: a target gene of miR‐15b

**Figure 4:**
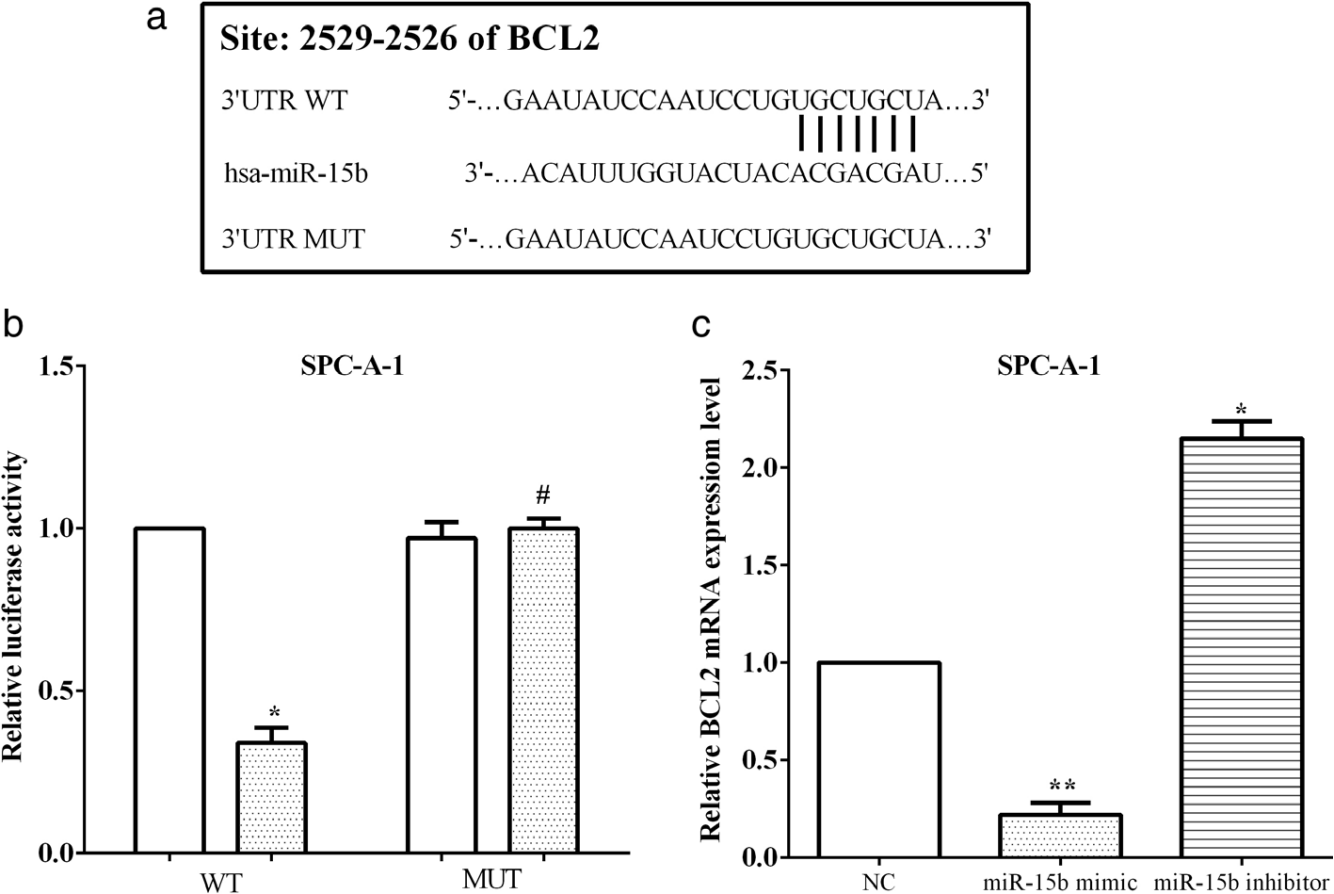
BCL2 was a target gene of miR‐15b. (**a**) TargetScan predicted BCL2 was a target of miR‐15b. (**b**) MiR‐15b mimic reduced the luciferase activity of cells that transfected BCL2 mRNA 3′‐UTR. (**c**) The expression of BCL2 was regulated by miR‐15b in SPC‐A1 cells. (**b**) (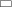) NC, (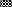) miR‐15b mimic; (**c**) (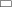) NC, (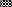) miR‐15b mimic, (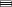) miR‐15b inhibitor.

The potential target genes of miR‐15b were predicted by TargetScan, and BCL2 was predicted to be a target of miR‐15b. To validate that BCL2 was a direct target of miR‐15b, we mutated the predicted sequences, and performed a dual luciferase reporter assay (Fig 4a). SPC‐A1 cells were cotransfected with pmirGlo‐BCL2‐WT (WT) or pmirGlo‐BCL2‐MUT (MUT) and the miR‐15b mimic or negative control. The miR‐15b mimic reduced the luciferase activity of cells that transfected wild‐type 3′‐UTR of BCL2 mRNA (*P* < 0.05), while it did not alter the activity of mutated 3′‐UTR of BCL2 mRNA (*P* > 0.05) (Fig 4b). The expression of BCL2 was calculated in SPC‐A1 cells after transfection with the miR‐15b mimic or inhibitor using RT‐qPCR, and we discovered that BCL2 was reduced by transfection of the miR‐15b mimic (*P* < 0.01), whereas transfection of the miR‐15b inhibitor increased the expression of BCL2 in SPC‐A1 cells (*P* < 0.05) (Fig 4c).

### Expression of BCL2 in LUAD

**Figure 5:**
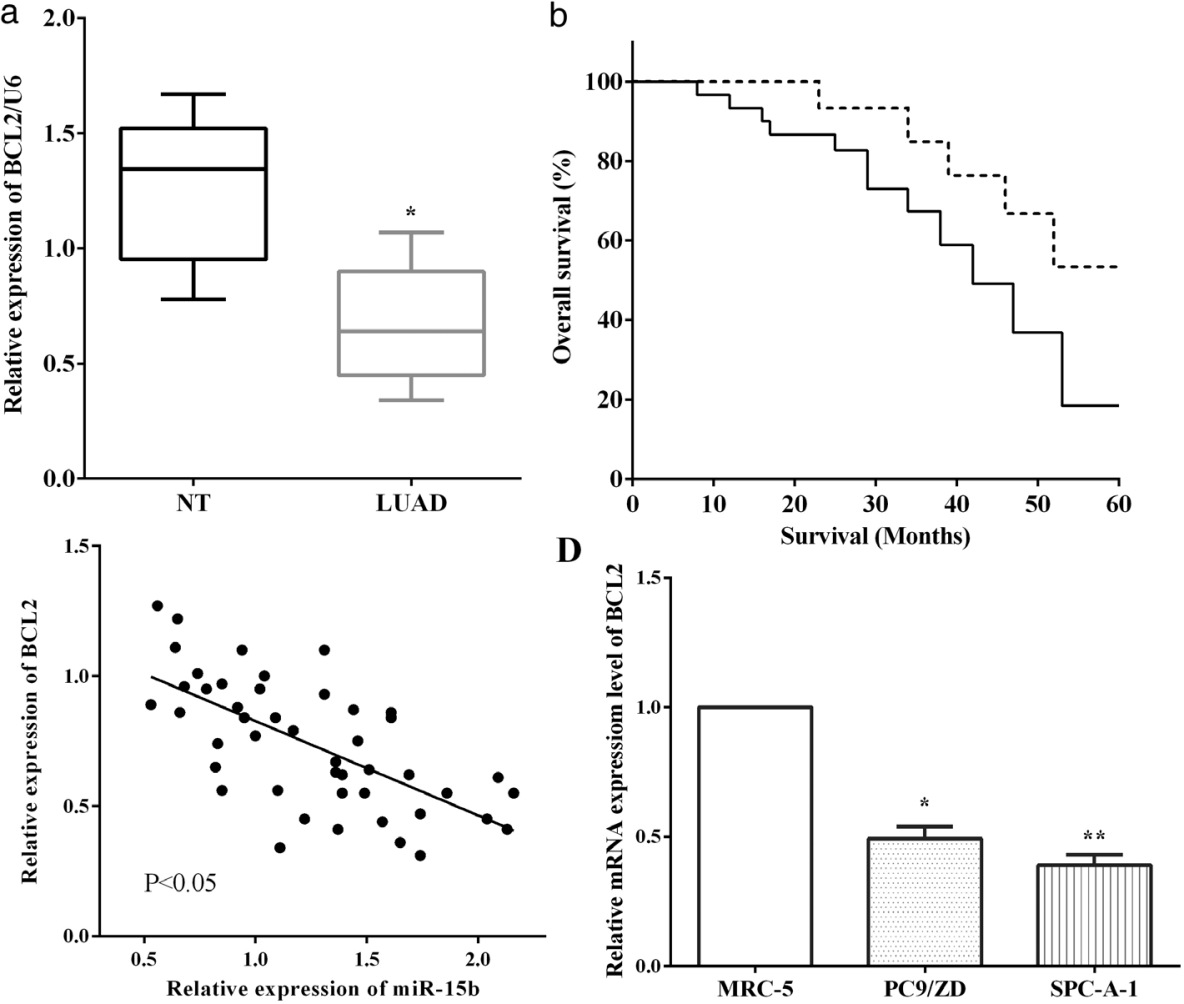
The expression of BCL2 in LUAD. (**a**) BCL2 expression was low in LUAD tissues compared with adjacent normal lung tissues. (**b**) Downregulation of BCL2 was associated with poor prognosis. (**c**) miR‐15b negatively regulated BCL2 in LUAD tissues. (**d**) BCL2 was remarkably reduced in PC9/ZD and SPC‐A1 cells compared to the normal cell line MRC‐5. (**b**) (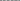) BCL2(+), (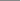) BCL2(−).

The expression of BCL2 in tissues and cell lines was evaluated by RT‐qPCR. There was a low expression of BCL2 in LUAD tissues compared with the adjacent normal lung tissues (*P* < 0.05) (Fig 5a). Moreover, the Kaplan‐Meier method was applied to measure the five‐year overall survival, and we found that downregulation of BCL2 was associated with a poor prognosis (*P* < 0.05) (Fig 5b). Subsequently, the connection between the expression of miR‐15b and BCL2 in LUAD tissues was calculated. As we expected, miR‐15b had a negative connection with BCL2 in LUAD tissues (*P* < 0.05) (Fig 5c). Moreover, the expression of BCL2 was also assessed in cell lines, and we discovered that BCL2 was remarkably reduced in PC9/ZD (*P* < 0.05) and SPC‐A1 (*P* < 0.01) cells compared to normal cell line MRC‐5 (Fig 5d).

### Knockdown of BCL2 suppressed cell proliferation and migration

**Figure 6:**
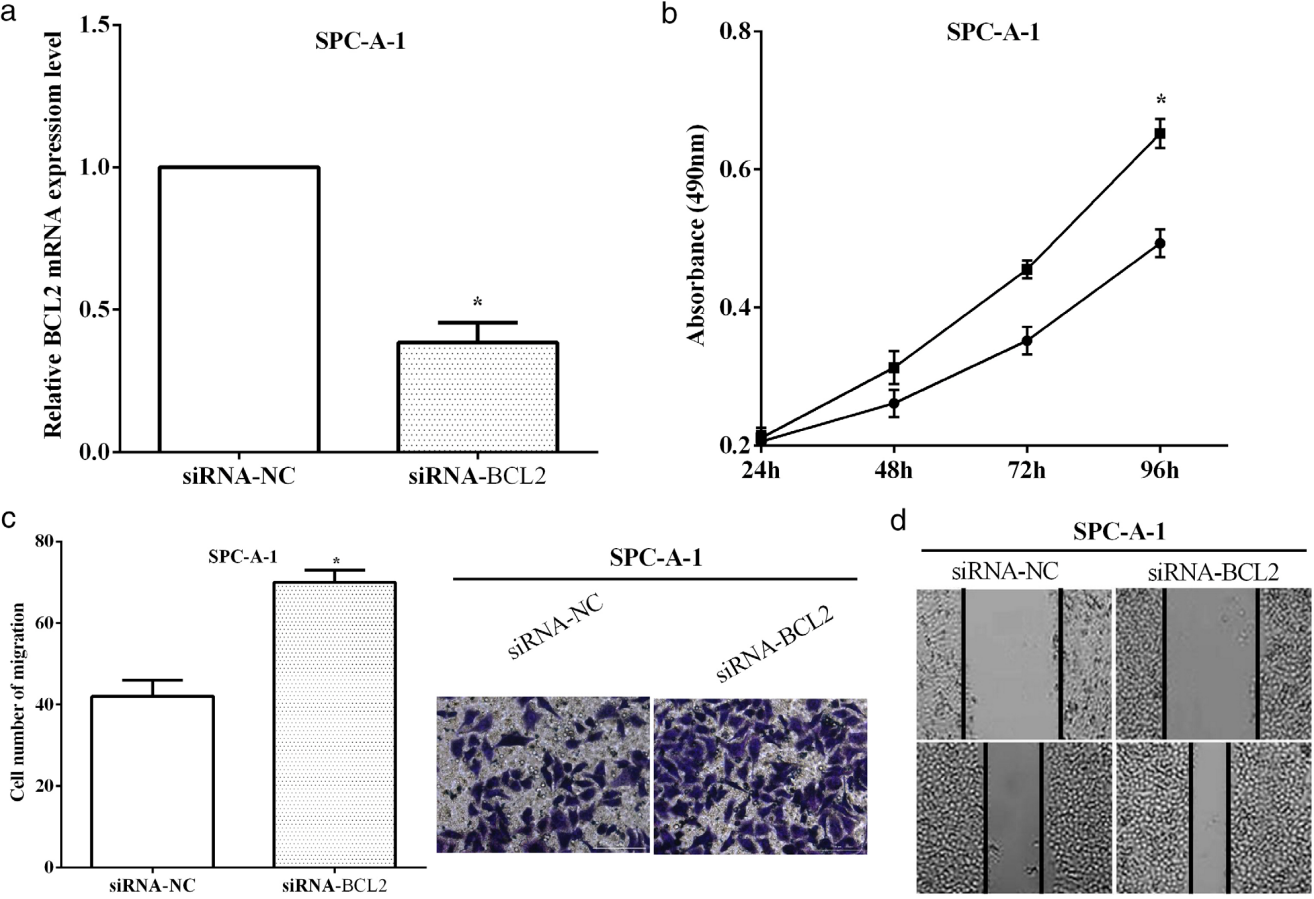
Knockdown of BCL2 suppressed cell proliferation and migration. (**a**) The transfection efficiency of knockdown of BCL2 by RT‐qPCR. (**b**) CCK‐8 assay revealed that cell proliferation was promoted by knockdown of BCL2. (**c**) Transwell assay demonstrated that cell migration was enhanced after interference of BCL2. (**d**) Wound healing assay revealed that knockdown of BCL2 promoted cell migration. (**b**) (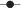) siRNA‐NC, (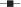) siRNA‐BCL2.

In SPC‐A1 cells, BCL2 was knocked down by siRNA‐BCL2 and the transfection efficiency was evaluated by RT‐qPCR (*P* < 0.05) (Fig 6a). CCK‐8 assay revealed that cell proliferation was promoted by BCL2 knock down (*P* < 0.05) (Fig 6b). Transwell assay demonstrated that cell migration was enhanced after interference of BCL2 (*P* < 0.05) (Fig 6c). Wound healing assay indicated that knockdown BCL2 enhanced cell migration (Fig 6d).

### BCL2 partially reversed the role of miR‐15b in LUAD cells

**Figure 7:**
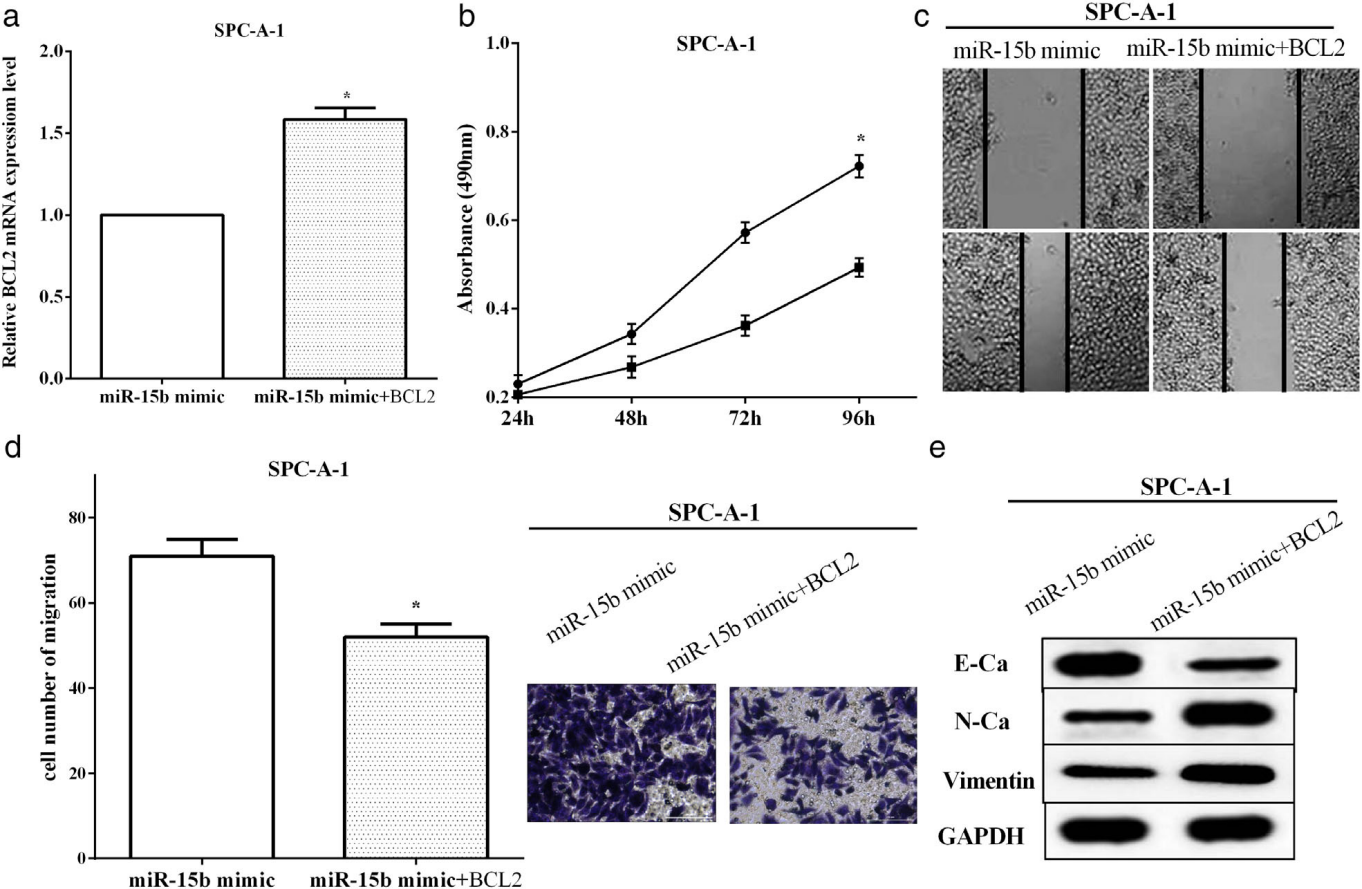
BCL2 partially reversed the role of miR‐15b in LUAD cells. (**a**) pcDNA3.1‐BCL2 plasmid transfected into miR‐15b overexpressed SPC‐A1 cells. (**b**) Cell proliferative ability was enhanced by overexpressing BCL2. (**c**, **d**) Cell migration was increased after transfection of pcDNA3.1‐BCL2 versus cells only transfected with miR‐15b mimic. (**e**) BCL2 partially reversed the role of miR‐15b on LUAD cells and epithelial‐mesenchymal transition (EMT). (**a**) (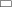) miR‐15b mimic, (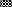) miR‐15b mimic+BCL2; (**b**) (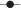) miR‐15b mimic, (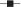) miR‐15b mimic+BCL2; (**d**) (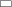) miR‐15b mimic, (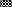) miR‐15b mimic+BCL2.

To explore the role of BCL2 in miR‐15b mimic‐transfected cells, we utilized pcDNA3.1‐BCL2 plasmid transfected into miR‐15b overexpressed SPC‐A1 cells to overexpress BCL2 (*P* < 0.05), as demonstrated in Fig 7a. CCK‐8 and Transwell assays were employed to evaluate the proliferative and migratory capacities in SPC‐A1 cells. Cell proliferative ability was enhanced by overexpressing BCL2 (*P* < 0.05) (Fig 6b). Wound healing and Transwell assays revealed that cell migration was increased after transfection with pcDNA3.1‐BCL2, versus cells only transfected with miR‐15b mimic (*P* < 0.05) (Fig 7c,d). The EMT associated proteins were assessed using western blot, and we found that overexpression of BCL2 enhanced N‐cadherin and vimentin expression, while it impaired the expression of E‐cadherin (Fig 7e). All the results elucidated that BCL2 partially reversed the role of miR‐15b on LUAD cell proliferation, migration and EMT.

## Discussion

Lung adenocarcinoma is a subtype of NSCLC that accounts for 40% of total LC.^28^ The morbidity and mortality of LUAD patients is still poor despite the advances in treatment.^29^ Therefore, the verification of novel molecular markers for early diagnosis and therapy of LUAD patients is urgently required.

MiR‐15b has been reported to promote cell viability, migration, invasion and EMT in gastric cancer.^30^ We discovered that miR‐15b was overexpressed in LUAD tissues and cells. Moreover, upregulation of miR‐15b has been reported to be associated with poor prognosis in patients with hepatocellular carcinoma.^31^ In this study, we found that overexpression of miR‐15b predicted a worse outcome for LUAD patients, which was consistent with the findings above. Downregulation of miR‐15b has been reported to inhibit cell migration and metastasis in colorectal cancer.^32^ Similarly, in NSCLC, miR‐15b enhanced cell proliferation and invasion.^33^ Our findings were consistent with all these results, and we determined that miR‐15b improved cell viability, migration and EMT in LUAD and miR‐15b enhanced LUAD cell growth in vivo.

In several studies, BCL2 has been reported to act as a target gene of multiple miRNAs, including miR‐153, miR‐136 and miR‐365.^34^, ^35^, ^36^ BCL2 has also been elucidated to be a target gene of miR‐15b in condylar hyperplasia and liver cancer.^37^, ^38^ miR‐15b has been shown to play a role in the development of multidrug resistance in gastric cancer cells, at least in part by modulation of apoptosis via targeting BCL2.^39^ Consistent with all these findings, we discovered that BCL2 was a target gene of miR‐15b in LUAD, and its expression was mediated by miR‐15b through targeting to its mRNA 3′‐UTR. In addition, BCL2 reversed functions of miR‐15b on promoting cell proliferation, migration and EMT in SPC‐A1 cells.

In conclusion, MiR‐15b was overexpressed in LUAD and miR‐15b overexpression predicted a worse outcome for LUAD patients. MiR‐15b promoted cell viability, migration and EMT through inhibiting BCL2 expression by targeting to its mRNA 3′‐UTR. BCL2 partially reversed functions of miR‐15b on promoting cell proliferation, migration and EMT in SPC‐A1 cells. MiR‐15b exerts carcinogenesis by inhibiting the expression of BCL2, which means that regulating miR‐15b expression may also be a promising therapeutic strategy for LUAD. In future, the development of drugs similar to miR‐15b may have important implications in the treatment of LUAD.

## Disclosure

The authors declare that there are no conflicts of interest.
